# Visualizing Active Enzyme Complexes Using a Photoreactive Inhibitor for Proximity Ligation – Application on γ-Secretase

**DOI:** 10.1371/journal.pone.0063962

**Published:** 2013-05-24

**Authors:** Sophia Schedin-Weiss, Mitsuhiro Inoue, Yasuhiro Teranishi, Natsuko Goto Yamamoto, Helena Karlström, Bengt Winblad, Lars O. Tjernberg

**Affiliations:** KI-Alzheimer Disease Research Center (KI-ADRC), Karolinska Institutet, Department of Neurobiology, Care Sciences and Society (NVS), Novum Level 5, Stockholm, Sweden; University of Maastricht (UM), The Netherlands

## Abstract

Here, we present a highly sensitive method to study protein-protein interactions and subcellular location selectively for active multicomponent enzymes. We apply the method on γ-secretase, the enzyme complex that catalyzes the cleavage of the amyloid precursor protein (APP) to generate amyloid β-peptide (Aβ), the major causative agent in Alzheimer disease (AD). The novel assay is based on proximity ligation, which can be used to study protein interactions in situ with very high sensitivity. In traditional proximity ligation assay (PLA), primary antibody recognition is typically accompanied by oligonucleotide-conjugated secondary antibodies as detection probes. Here, we first performed PLA experiments using antibodies against the γ-secretase components presenilin 1 (PS1), containing the catalytic site residues, and nicastrin, suggested to be involved in substrate recognition. To selectively study the interactions of active γ-secretase, we replaced one of the primary antibodies with a photoreactive γ-secretase inhibitor containing a PEG linker and a biotin group (GTB), and used oligonucleotide-conjugated streptavidin as a probe. Interestingly, significantly fewer interactions were detected with the latter, novel, assay, which is a reasonable finding considering that a substantial portion of PS1 is inactive. In addition, the PLA signals were located more peripherally when GTB was used instead of a PS1 antibody, suggesting that γ-secretase matures distal from the perinuclear ER region. This novel technique thus enables highly sensitive protein interaction studies, determines the subcellular location of the interactions, and differentiates between active and inactive γ-secretase in intact cells. We suggest that similar PLA assays using enzyme inhibitors could be useful also for other enzyme interaction studies.

## Introduction

γ-Secretase has been extensively studied as it catalyzes the final step in generation of the neurotoxic amyloid β-peptide (Aβ), which is involved in the development of Alzheimer disease (AD) [1]. It is composed of the four protein subunits presenilin 1 (PS1) or 2 (PS2), nicastrin, anterior pharynx-defective phenotype 1 (Aph-1) and PS-enhancer 2 (Pen-2). PS1 and PS2 contain nine transmembrane (TM) domains [2] of which TM regions six and seven contain two well-conserved aspartyl residues that are required for γ-secretase activity [3], [4]. Nicastrin is a type 1 TM protein containing a large and highly glycosylated ectodomain [5] and several studies indicate that nicastrin is involved in substrate selection [6], [7]. In γ-secretase assembly, nicastrin first binds to the seven TM protein Aph1, believed to be involved in stabilization and scaffolding [8], followed by the addition of PS to the first subcomplex. Finally, the relatively small protein, Pen-2 (containing two TM domains), joins the complex and facilitates auto-proteolytic cleavage of PS to generate an N-terminal (NTF) and a C-terminal fragment (CTF), which is required to generate active γ-secretase [9]. Since γ-secretase is a large TM enzyme with many components and a catalytic site embedded in the middle of the membrane [10], structure-function studies are difficult and the reports presented so far are few. Low resolution structures have been determined by electron microscopy [11], [12], [13], but crystallography data is still lacking. Method development is thus important to elucidate the structure/function of γ-secretase. Knowledge about the subcellular location of active γ-secretase could for instance be used for subcellular targeting of the active enzyme.

Aspartyl protease transition state analogue inhibitors are useful tools for functional studies of γ-secretase. One such compound is L-685,458, which potently inhibits γ-secretase activity [14], [15] and signal peptide peptidase [16]. Our group previously designed an L-685,458-based compound for the efficient affinity purification of γ-secretase and its interacting proteins [17]. The compound, denoted GCB (γ-secretase inhibitor with a cleavable biotin group) contained L-685,458 coupled to a long hydrophilic linker connected to a disulphide bond and a biotin group. In the present study, we designed a similar compound that additionally contains a photoreactive group enabling covalent linkage to nearby components, called GTB (γ-secretase inhibitor with a transferable biotin group). We characterized this compound and developed a method based on proximity ligation, in which we used GTB to visualize active γ-secretase in neurons. In situ proximity ligation assay (PLA) is a method used for highly sensitive protein-protein interaction studies [18]. The sample (fixed and permeabilized cells or tissue sections) is usually incubated with two primary antibodies recognizing the interacting proteins, followed by secondary antibodies bound to different oligonucleotide strands. If these strands are in proximity they can be ligated, amplified by a rolling circle mechanism and fused to complementary fluorescently labelled oligonucleotides. One pair of interacting proteins can thus be detected as a signal in a fluorescence microscope. Since antibodies cannot discriminate between immature and mature forms of γ-secretase, we developed an assay where we can specifically detect interactions only with the mature form. By replacing one primary antibody with GTB and the corresponding detection probe with oligonucleotide-conjugated streptavidin and comparing this method with traditional PLA, we were able to differentiate between inactive and active γ-secretase in situ.

## Materials and Methods

### Synthesis of GTB

**Figure 1:**
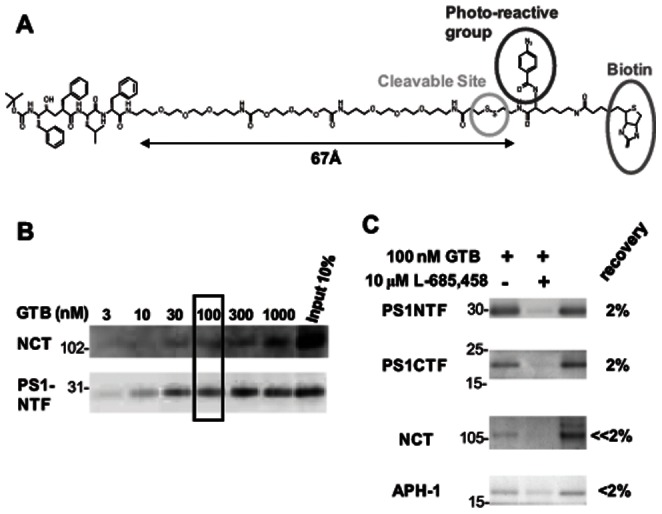
Structure and characterization of GTB. (A) Structure of the γ-secretase inhibitor with a transferable biotin group, denoted GTB, designed with a PEG linker, a disulfide bond, a photoreactive group and a biotin group. The distance between the inhibitor part and the biotin moiety was determined by 3D ChemDraw (CambridgeSoft). (B) Solubilized γ-secretase prepared from rat brain was incubated with different concentrations of GTB and isolated with streptavidin beads. The captured γ-secretase complex was eluted by SDS sample buffer and subjected to western blot for the indicated γ-secretase subunit. (C) Solubilized γ-secretase prepared from rat brain was incubated with 100 nM GTB in the presence (+) or the absence (-) of 10 µM L-685,458 and isolated with streptavidin beads and analyzed as described above.

GTB was designed in our lab and synthesized by Chemilia AB (Huddinge, Sweden). It is composed of γ-secretase inhibitor L685,458 that has been attached to a hydrophilic PEG linker, a disulfide bond, a photoreactive group and a biotin group (Figure 1A). The synthesis of the methyl ester of the L-685,458 acid derivative was described previously [19]. The L-685,458 derivative was reacted with 10 equivalents of diamido-dPEG diamine (eChemShop, Newark, DE, USA) under EDC (N-(3-Dimethylaminopropyl)-N′-ethylcarbodiimide hydrochloride) and 1-hydroxybenzotriazole hydrate overnight. The resulting product was treated with ProFound Label Transfer Sulfo SBED Protein (1 equiv, Pierce) overnight. The reaction mixture was purified by silica gel chromatography to give GTB.

### Antibodies, Proteins and Reagents

The following antibodies were used: Mouse monoclonal anti-PS1 loop (MAB5232, Millipore) raised against amino acids 263–378 of human PS1 (referred to as PS1-CTF); Rabbit polyclonal anti PS1-NTF (529591, Calbiochem, Darmstadt, Germany) raised against amino acid residues 1–65 of human PS1; Monoclonal mouse anti-nicastrin (MAB 5556, Millipore) raised against the 18 C-terminal amino acids of mouse nicastrin (referred to as mouse nicastrin-CT); Rabbit polyclonal anti-nicastrin (Abcam ab 24741) recognizing N-terminal amino acids 103–124 of nicastrin (referred to as nicastrin-NT); Nicastrin (N1660, Sigma, St. Louis, MO, USA) raised against C-terminal residues 693–709 of human nicastrin (referred to as human nicastrin-CT); Aph-1aL (PRB-550P; COVANCE, Berkeley, CA, USA), raised against the C-terminal region of human Aph-1aL; UD1 raised against the N-terminal residues ERVSNEEKLNL of Pen-2 (a gift from Dr. Jan Näslund, Karolinska Institutet); Rabbit polyclonal anti-HM13 (PROTEINTECH group, Manchester, England). Secondary antibodies used as PLA probes (PLA probe MINUS and PLA probe PLUS) were purchased from Olink Bioscience. Ultrapure streptavidin was from AppliChem GmbH (Darmstadt, Germany). Labeling of streptavidin with oligonucleotides to generate directly conjugated PLA probes was done with the Duolink in situ probemaker plus or minus kits (Olink Bioscience), according to the manufactureŕs instructions. Blocking solution, antibody diluent, probe diluent, wash buffers, detection reagents and mounting medium were purchased from Olink Bioscience. Atto 594 streptavidin was purchased from from ATTO-TEC Gmbh, Siegen, Germany. Alexa Fluor® 555 phalloidin for the staining of actin was from Invitrogen. γ-Secretase inhibitor L-685,458 was from Sigma-Aldrich. Signal peptide peptidase (HM13) inhibitor (Z-LL)2 ketone was from Merck.

### Culturing of Cell Lines

Blastocyst-derived embryonic stem cells deficient for PS1 and PS2 (BD8 cells), cells with one allele PS1 (BD3 cells) [20] and cells stably expressing PS1 (BD8-PS1 cells) [21], were cultured in ES medium; Dulbecco’s modified Eagle’s medium (DMEM) supplemented with 10% (v(v) fetal calf serum, 1 mM sodium pyruvate, 0.1mM β-mercaptoethanol, and nonessential amino acids (Invitrogen).

Nicastrin deficient mouse embryonic fibroblasts (Nct−/−MEF) cells and wild type MEF cells (Nct +/+ MEF) [22], were cultured in Dulbecco’s modified Eaglés medium (DMEM) supplemented with 10% (v/v) fetal bovine serum (Invitrogen).

### γ-Secretase Activity Assay

γ-Secretase activity was measured as described before [17]. Briefly, CHAPSO-solubilized membranes isolated from blastocyst-derived ES-cells deficient in PS1 and PS2, stably expressing PS1 (BD8-PS1 cells) [21] were incubated in the absence or presence of L-685,458 or GTB for 16 h at 37°C. The reaction was stopped by adding RIPA and boiling for 5 min. The samples were centrifuged at 10 000×g, and Aβ40 in the supernatants was measured by ELISA (Wako Chemicals, Osaka, Japan). Background, defined as the signal in the presence of 10 µM L-685,458, was subtracted. IC50 (nM) values were calculated using the GraphPad Prism 4.02 software (GraphPad Software, La Jolla, CA, USA).

### Preparation of Solubilized γ-secretase and Affinity Pulldown

Microsomal membranes were prepared from Sprague-Dawley rats and solubilized as described previously [17]. The samples were incubated with streptavidin-conjugated magnetic beads (Invitrogen) to remove endogenous biotinylated proteins for 16 h at 4°C. The supernatants were recovered by centrifugation at 1 000×g for 5 min and used as starting material (input) for pull-down experiments. The samples were incubated in the absence or presence of 10 µM L-685,458 as a competing inhibitor for 10 min at rt and then incubated with 100 nM GTB for 30 min at rt. The samples were irradiated on ice for 10 min by using a B100a Lamp at a distance of 7 cm. Five times concentrated RIPA was added to the samples to give a final 1 time RIPA concentration. The labeled samples were incubated with streptavidin-conjugated magnetic beads that were pre-equilibrated in RIPA for 2 h at 4°C. The resin was washed 3 times with RIPA. The labeled γ-secretase components were eluted from the resin by boiling for 3 min in the Tricine-SDS sample buffer.

### SDS-PAGE and Western Blot

SDS-PAGE and western blot (WB) was done as described previously [17]. Briefly, proteins were separated on 10–20% Tricine gels, Invitrogen, Carlsbad, CA, USA), transferred to PVDF membranes (Bio-Rad, Hercules, CA, USA) and then probed with specific antibodies. Immune complexes were visualized by SuperSignal West Dura enhanced chemiluminescence reagent (Pierce, Rockford, IL, USA). Hyperfilm ECL (GE Healthcare, Piscataway, NJ, USA) was used for exposure, and exposed films were scanned using an AGFA Duoscan. For quantification, a ChemiDoc CCD camera system (Bio-Rad) was used. Bands were quantified using the Quantity One analysis software Version 4.5.2 (Bio-Rad). The density of the bands was calculated as a percentage of a standard (input sample) run on each gel.

### Culturing, Fixation and Permeabilization of Mouse Primary Hippocampal Neurons

Neurons from mouse brain hippocampus were cultured on poly-D-lysine coated glass bottom culture dishes P35G-0-10-C (MatTek corporation, Ashland, USA), essentially as described previously [23]. Cortex cells were added to supply growth factors required for the hippocampal cell growth. The cortex cells were therefore first attached to the outer portion plastic of the plates. Hippocampal cells were subsequently seeded on the inner 10 mm microwell. The medium was changed to neurobasal medium containing 2% B27 (Invitrogen) and 1% L-glutamine (Invitrogen) and the cells were grown at 37°C in a cell incubator (humidified, 5% CO_2_) for 14 days. After culturing, the cells were washed in DPBS and fixed with 4% formaldehyde (Sigma, HT5011) for 10 minutes at 4°C. The fixed cells were washed in DPBS and either used for PLA directly or stored at 4°C.

### GTB Immunocytochemistry

Unless otherwise stated, incubations were done at rt. HEK/APP cells were cultured as described before [24], washed with PBS and fixed with 4% Formaldehyde (Sigma, HT5011) for 10 min at 4°C. HEK/APP cells or mouse primary hippocampal neurons were permeabilized with 0.4% CHAPSO in PBS for 10 min, blocked with avidin for 15 min, washed with PBS, blocked with biotin for 15 min, washed with PBS and blocked with 3% normal goat serum (NGS) in the absence or presence of L-685,458 (10–20 µM) for 30 min at 37°C, incubated with GTB (100, 200 or 400 nM) in the absence or presence of L685,458 in 3% NGS for 30–60 min at 37°C in darkness, followed by irradiation on ice for 10 min by using a B100a Lamp at a distance of 7 cm. The cells were then washed with PBS, incubated with streptavidin-Alexa 488 (1∶500) or Atto-594-streptavidin in 3% NGS for 30 min at 37°C, washed with PBS and mounted with mounting medium containing DAPI.

### PLA Conditions

The confocal plates that the mouse primary hippocampal neurons were cultured in were used as a reaction chamber for the entire PLA assays. The protocol suggested by Olink Bioscience for Duolink II solutions was essentially followed. The following basic procedures were the same for all assays; Immediately prior to the PLA assay, the cells were permeabilized in 0.4% CHAPSO for 10 min at rt. The cells were then incubated with blocking solution (Olink Bioscience) for 30 min at 37°C. Then, the cells were incubated with two primary antibodies or one antibody & GTB for 1 h at 37°C. After washing with Duolink washing solution A, the cells were incubated with secondary antibodies conjugated to oligonucleotide strands denoted MINUS and PLUS (PLA probes MINUS and PLUS, respectively, Olink Bioscience) or one PLA probe and one streptavidin conjugated to oligonucleotide PLUS or MINUS. After washing with washing solution A, the incubation steps with detection reagents (Detection reagents far red, Olink Bioscience) were done according to the manufacturers instructions, with a ligation step and a polymerization step, both conducted at 37°C. All incubation steps at 37°C were conducted in a humid chamber, i.e. plastic box with wet tissue papers placed at the bottom.

### Validation of Antibodies with PLA

The antibodies used for PLA were validated with the proximity ligation assay, using a single recognition setup. The unlabeled primary antibodies were used in the first incubation step, followed by one PLA probe MINUS and one PLA probe PLUS, both directed to the same primary antibody. The remaining steps followed the basic procedure described above.

### PLA with Antibodies for Protein-protein Interaction Studies

The interaction between nicastrin and PS1 was first studied with a double recognition PLA setup using PS1-NTF antibody, mouse nicastrin-CT antibody and the PLA probes anti-mouse PLUS and anti-rabbit MINUS. The remaining steps followed the basic procedure described above.

### Proximity Ligation with GTB

To specifically detect protein interactions for active γ-secretase, GTB, which binds to the active site of γ-secretase and thus excludes the detection of immature γ-secretase, was used in a PLA assay. In this assay, the biotin group on GTB was probed by oligonucleotide-labeled streptavidin. To avoid unspecific staining by the streptavidin probe in the cells, an extra blocking step was therefore added prior to the blocking step with the Olink blocking solution. In this additional blocking procedure, the cells were incubated with avidin for 15 min at rt, washed twice with PBS, incubated with biotin for 15 min and then washed twice with PBS again. After both blocking steps, the cells were incubated with 200 nM GTB and primary antibody directed to either PS1 or nicastrin in the first incubation step. As a negative control for this assay, 50-fold excess (10 µM) of the inhibitor L-685,458, was used both in the blocking solution and in the first incubation. Directly after this first incubation, the reaction chambers were put on ice and irradiated for 10 min by using a B100a Lamp at a distance of 7 cm. In the second incubation step, the cells were incubated with streptavidin conjugated to oligonucleotide PLUS (as a probe for the bound GTB) and Duolink probe (anti-mouse or anti-rabbit MINUS) to detect the PS1 or nicastrin, diluted in probe diluent (Olink Bioscience). The remaining steps followed the basic procedure described above.

### Image Visualization and Processing

The samples were examined using a laser scanning confocal microscope (LSM 510 META, ZEISS) with the Plan Apochromatic 63x/1.4 Oil or 40x/1.3 oil immersion objectives. PLA signals were quantified with the Duolink Image Tool software (Olink Bioscience). All proximity ligation experiments were repeated 3–4 times and in each experiment the PLA signals were calculated in 3–5 neurons in each preparation.

## Results

The purpose of this study was to develop a highly sensitive assay to visualize the subcellular location and interactions for active γ-secretase in situ. To this end, we designed a compound, denoted GTB, which contains the γ-secretase inhibitor L-685,458, a PEG linker containing a disulfide bond, a photoreactive group and a biotin group. The latter groups make the inhibitor accessible to ligands and reagents for proximity ligation.

### Characterization of GTB

GTB is similar to GCB, which was characterized previously [17], with the difference that a photoreactive group has been inserted in GTB. This group is useful in assays with extensive washing steps, since it becomes covalently bound to nearby molecules when subjected to UV light, and thus cannot be washed off. The chemical structure of GTB is shown in Fig. 1A. The inhibitor part and the photoreactive group are maximally around 67Å apart in GTB, as determined by 3D ChemDraw. The inhibitory potency of GTB, determined by measuring Aβ40 production in microsomal membranes from BD8-PS1 cells by ELISA, was similar as for L-685,458 and GCB with an IC50 of 11.3 nM as compared to 10.3 nM for L-685,458 and 13.1 nM for GCB [17].

### Affinity Purification of γ-secretase Components by GTB

γ-Secretase components were affinity purified from rat microsomal membranes by using GTB in a similar manner as described previously for GCB [17], using streptavidin-conjugated magnetic beads (Invitrogen) to capture the bound proteins after UV irradiation. The GTB concentration was first varied from 3 to 1000 nM and the pulldown efficacy was evaluated with western blot for nicastrin and PS1-NTF. A dose dependency was observed (Fig. 1B) and saturation was approached at 100–300 nM. PS1-NTF, PS1-CTF, nicastrin and Aph-1, but not Pen-2, were subsequently found to be pulled down by this procedure and the specificity of GTB was confirmed by competition using excessive concentration (10 µM) of the inhibitor L-685,458 at a fixed concentration (100 nM) of GTB (Fig. 1C). The lack of detection of Pen-2 may be due to the fact that it is a small molecule compared to the other γ-secretase components, which makes it less likely that GTB binds to Pen-2. Alternatively, Pen-2 may be located more distal from the active site than the other components. In our previous study describing the characterization of GCB, the washing condition was milder, 0.5% CHAPSO, since GCB is not covalently linked to γ-secretase. The recovery of the γ-secretase components by affinity purification was ∼10-fold lower with the GTB method, reflecting the fact that only the proteins that bound directly to GTB were detected.

### Evaluation of GTB Concentration by Immunocytochemistry

The concentration dependency for GTB binding to γ-secretase in fixed and permeabilized cells was evaluated by immunocytochemistry in HEK/APP cells and mouse primary hippocampal neurons. Three concentrations of GTB (100, 200 and 400 nM) were evaluated by using fluorescently labeled streptavidin for detection. 200 and 400 nM GTB showed similar staining, whereas 100 nM resulted in weaker staining (not shown). Thus, we concluded that 200 nM, which is similar to the optimal concentrations needed for affinity purification as well as for inhibition of Aβ production, is sufficient to obtain close to saturation binding of GTB to γ-secretase in the cells. The competitive effect of 10 µM L-685,458 in cells incubated with 200 nM GTB was confirmed (Figure S1).

### Evaluation of Antibodies with PLA and WB

**Figure 2:**
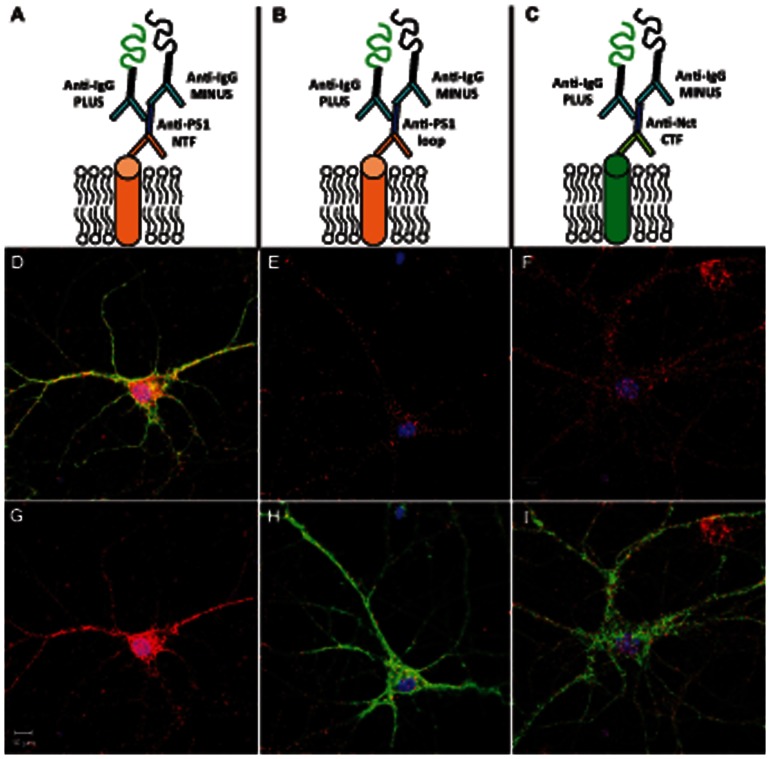
Antibody validation. The applicability of antibodies for PLA was validated by a single recognition PLA experiment, as described in Experimental Procedures and schematically outlined in Fig. 2A–C. In addition to the PLA signals (red dots), the cells (embryonic mouse primary hippocampal neurons) were stained with DAPI and phalloidin for nuclei (blue) and actin (green), respectively. DAPI and PLA staining are shown for the PS1 antibody recognizing the N-terminus, the PS1 antibody recognizing the cytosolic loop and the nicastrin antibody recognizing a cytosolic epitope in D, E and F, respectively. DAPI, PLA and phalloidin staining for the same antibodies are shown in G, H and I, respectively.

Two PS1 and two nicastrin antibodies were evaluated by PLA in a single recognition setup (Fig. 2A–C) in mouse primary hippocampal neurons. For all four antibodies, 100–200-fold dilutions were tested and gave similar results, indicating that saturation was obtained at these concentrations. The PS1-NTF (Fig. 2D and G), PS1-CTF (Fig. 2E and H) and nicastrin-CT antibodies (Fig. 2F and IJ) all gave a high extent of PLA signals throughout the entire neurons. Staining with the nicastrin-NT antibody gave only a few PLA signals (not shown) and thus, this antibody was excluded in the remaining experiments. In summary, the PS1-NTF, PS1-CTF and nicastrin-CT antibodies are appropriate to use for PLA experiments with mouse primary hippocampal neurons and PS and nicastrin are expressed throughout the entire neuron.

The specificities of the PS1 antibodies used for PLA were determined by WB in BD8 and BD3 cells and mouse primary hippocampal neurons. The PS1-CTF antibody gave only one band at ∼20 kDa in neurons and BD3 cells, and this band was not observed in BD8 cells. The PS1-NTF antibody showed one major band (>95% of the staining) at ∼30 kDa in BD3 cells and only one band with the same migration properties in the neurons. As expected, this band was not present in BD8 cells. Noteworthy, the band was undetectable after boiling the samples in sample buffer. The mouse nicastrin-CT antibody was tested by WB in Nct−/− MEF and Nct+/− MEF cells and mouse primary hippocampal neurons. Two bands were observed in the Nct+/− MEF cells, one at ∼110 and one at ∼130 kDa, that were not observed in the Nct−/− cells. Only one band at ∼130 kDa was observed in the neurons. The bands at 110 and 130 kDa presumably correspond to immature and mature nicastrin, respectively.

### PLA with Antibodies for Protein-protein Association Studies

**Figure 3:**
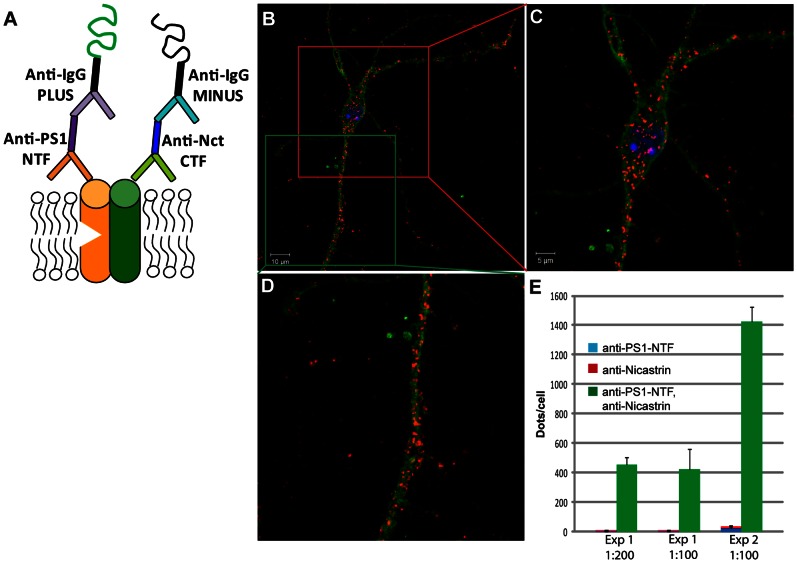
PLA with primary antibodies and oligonucleotide-conjugated secondary antibodies. The PLA assay was performed as depicted in Fig. 3A. Mouse nicastrin-CT and PS1-NTF antibodies were used as primary antibodies and anti-mouse PLUS and anti-rabbit MINUS (Olink Bioscience) were used as secondary antibodies. In addition to PLA (red dots), the cells (embryonic mouse primary hippocampal neurons) were stained with DAPI and phalloidin for nuclei (blue) and actin (green), respectively. An image of a neuron (B) and a close-up of the cell body (C) and neurites (D). The number of dots were quantified with Duolink Image Tool and expressed as fold increase to the sum of the negative controls (lacking either the PS1 or nicastrin antibody) (E). Mean values ± SE from at least three cells in each experiment are shown.

The association between PS1 and nicastrin was studied by using the PS1-NTF and nicastrin-CT antibodies according to the dual recognition PLA setup shown in Fig. 3A. The specificity of the PLA signals was confirmed by the lack of signals in BD8 cells lacking PS1 and PS2, compared to a substantial number of signals in BD3 cells containing one PS1 allele (Figure S2). PLA signals were observed throughout the entire neuron, from the cell body to the distal parts of the neurites (Fig. 3B). Some areas containing more dense staining than other areas were observed both in the cell body (Fig. 3C) and in the neurites (Fig. 3D). The result from quantification of the PLA signals is in this and subsequent sections referred to as fold increase, meaning the ratio for the PLA signals in cells incubated with both primary antibodies divided by the sum of signals in the two negative controls, each lacking one of the primary antibodies (Fig. 3E). The number of PLA signals in negative controls containing only the detection probes (i.e. lacking both primary antibodies) was minimal and, therefore, neglected in this and subsequent PLA experiments. As in the antibody evaluation described above, dilution ratios of 1∶100 or 1∶200 of the antibody gave similar results, showing that sufficient (saturating) concentrations were used. Although the total number of PLA signals varied between different experiments, the fold increase, ∼40-fold, was stable. Thus, we conclude that PLA with a dual recognition antibody setup can be used to study the association between PS1 and nicastrin in situ in neurons.

### PLA with GTB

**Figure 4:**
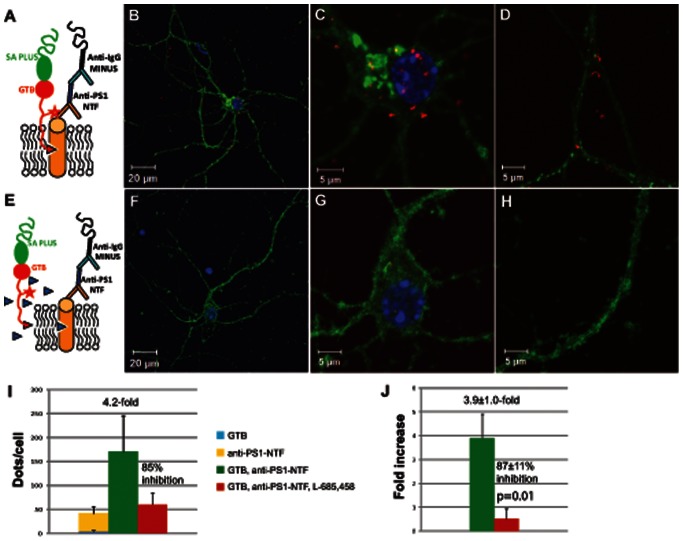
PLA with GTB and PS1-NTF antibody. PLA was done as schematically depicted in A in the absence (B, C and D) or as depicted in E in the presence(F, G and H) of competitor L-685,458. A neuron is shown (B and F), a close-up of the cell body from the corresponding neuron (C and G) and a selected part of the neurites (D and H). The number of dots were quantified with Duolink Image Tool and expressed as fold increase compared to the sum of the negative controls (lacking the PS1 antibody or GTB) and to the % inhibition in the presence of L-685,458, calculated in two different ways, as described in the results section (I and J). Mean values ± SE from at least three cells in three different experiments are shown. The p-value was calculated using one-tailed, paired t-test.

In order to study only active γ-secretase and not inactive subcomplexes, the proximity ligation method with GTB was developed. The method was evaluated by using PS antibodies and GTB in the absence or presence of L-685,458 according to the PLA setups shown in Fig. 4A and E. Although PS antibodies and GTB bind to the same protein, GTB binds to only active, i.e. cleaved PS, and therefore only the active form will give a signal with this setup. First, we evaluated the PLA assay using the PS1-NTF antibody and GTB (Fig. 4B-D). As negative controls, neurons were incubated with only GTB or only PS1-NTF antibody. Another type of negative control was introduced by the addition of the competitor L-685,458 in a 50-fold excess over the GTB concentration (Fig. 4F–H). As for PLA assays with only antibodies, the total number of PLA signals was highly variable in experiments conducted at different occasions despite the fact that the experiment parameters were kept the same. This could be due to many factors, for instance the size and shape of the cells grown in different batches as wells as the condition of the mouse that they were derived from. Importantly, the fold increase was stable when experiments conducted at different occasions were compared. The quantification data was performed in two different ways; i) The mean values of the number of PLA signals in the different experiments were first calculated and then the fold increase and % inhibition were calculated from that (Fig. 4I) or ii) The mean values of the fold increase and % inhibition in each experiment were directly calculated and then the mean values of fold increase and % inhibition between the experiments were calculated from that (Fig. 4J). The first way of calculating revealed a total number of 171 PLA signals, corresponding to 4.2-fold increase and 85% inhibition in the presence of L-685,458. The second way of calculating gave a 3.9±1.0 fold increase and 87±11% inhibition in the presence of L-685,458 for the PLA assay using the PS1-NTF antibody and GTB. The latter way of calculating enhanced the significance of the results by avoiding the variation in the total number of PLA signals between different experiments, whereas the first method takes into consideration the total amount of signals in the samples. Since both ways of calculating have advantages and disadvantages, the results from all proximity ligation experiments in this study are displayed in both ways. To conclude, the significance in the fold increase demonstrates the usefulness and specificity of GTB in the proximity ligation method. The extensive inhibition obtained by the competitor L-685,458 further supports this notion.

**Figure 5:**
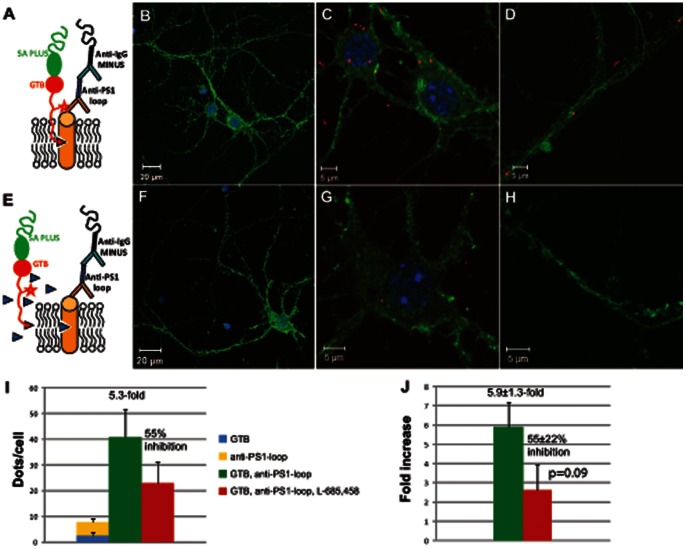
PLA with GTB and PS1-CTF antibody. PLA was done as schematically depicted in A in the absence(B, C and D) or, as depicted in E, in the presence(F, G and H) of competitor L-685,458. The entire neuron is shown (B and F), a close-up of the cell body from the corresponding neuron (C and G) and a selected part of the neurites (D and H). The dots were quantified with Duolink Image Tool and expressed as fold increase to the sum of the negative controls (lacking the PS1 antibody or GTB) and as % inhibition in the presence of L-685,458, calculated by two different ways, as described in the results section (I and J). Mean values ± SE from at least three cells in four different experiments are shown. The p-value was calculated using one-tailed, paired t-test.

Next, a similar PLA setup as described above was conducted, except that the PS1-CTF antibody was used instead of the PS1-NTF antibody (recognizing the cytosolic loop of PS1, Fig. 5A and E). Images of PLA with the PS1-CTF antibody and GTB in the absence (Fig. 5B–D) or presence (Fig. 5F–H) of L-685,458 are shown. The first way of calculating gave 41 signals/cell, corresponding to a 5.3-fold increase. Fifty-five percent of these signals were inhibited in the presence of L-685,458 (Fig. 5I). The second way of calculating (Fig. 5J) gave 5.9±1.3-fold increase and 55±22% inhibition in the presence of L-685,458. The total number of dots was thus higher for the PS1-NTF antibody (171/cell) than for the PS1-CTF antibody (41/cell) and the fold increase using the PS1-CTF antibody (5.9±1.3) was somewhat higher as compared to the value obtained with the PS1-NTF antibody (3.9±1.9). However, the inhibition by L-685,458 was higher for the PS1-NTF antibody (87±11%) than for the PS1-CTF antibody (55±22%). Thus, the PS1-NTF antibody is more suitable in this assay, presumably because the PS1-NTF antibody has higher affinity. Alternatively, the PS1-CTF antibody, which binds to the cytosolic loop of the CTF, might sterically hinder the binding of GTB or affect the conformation of the active site.

Since L-685,458 also has been reported to bind to SPP, we determined whether such interactions could affect our data. We first performed a proximity ligation assay using GTB and an SPP (HM13) antibody. The PLA signals observed were only few compared to the signals obtained with PS1 antibodies and they were fully competed in the presence of the SPP-specific inhibitor (Z-LL)2-ketone. Moreover, the presence of (Z-LL)2-ketone did not affect the proximity ligation assay using PS1-CTF antibody and GTB, confirming that GTB binding to SPP does not interfere with our novel assay. This is in accordance with our expectations, since SPP has not been reported to interact with γ-secretase.

**Figure 6:**
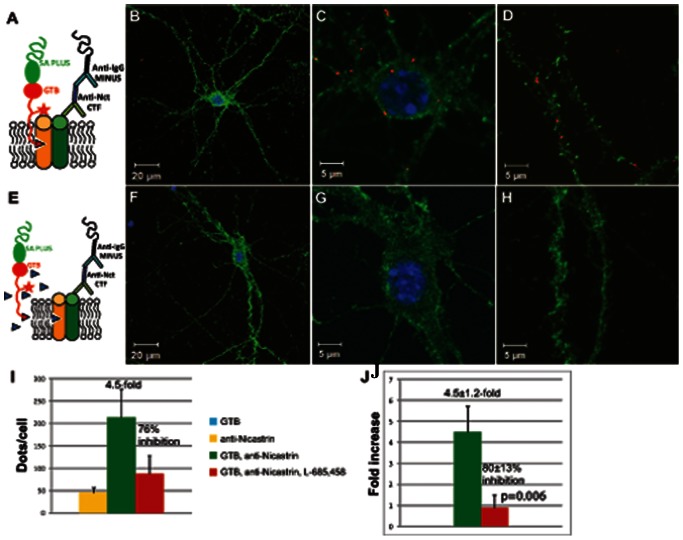
PLA with GTB and nicastrin antibody. PLA was done as schematically depicted in Fig. A and E. Mouse nicastrin-CTF antibody was used with GTB in the absence(B, C and D) or presence(F, G and H) of the competitor L-685,458. The entire neuron is shown (B and F), a close-up of the cell body from the corresponding neuron (C and G) and a selected part of the neurites (D and H). The PLA signals (red dots) were quantified with Duolink Image Tool and expressed as the ratio between the sample and the sum of the negative controls (lacking the nicastrin antibody or GTB) and as % inhibition in the presence of L-685,458, calculated by two different ways, as described in the results section (I and J). Mean values ± SE from at least three cells in three different experiments are shown. The p-value was calculated using one-tailed, paired t-test.

Finally, the association between nicastrin and the γ-secretase active site was studied by using GTB and the mouse nicastrin-CT antibody, according to the proximity ligation setup shown in Fig. 6A and E. Images from reactions in the absence (Fig. 6B–D) or presence (Fig. 6F–H) of L-685,458 are shown. The quantification of PLA signals was done as described above. The first way of calculating gave 214 signals/cell, corresponding to a 4.5-fold increase and a 76% inhibition (Fig. 6I). Calculating the second way gave 4.5±1.2-fold increase and 80±13% inhibition in the presence of L-685,458 (Fig. 6J). The values were thus similar to those for the PLA assay using the PS1-NTF antibody and GTB (3.9±1.0-fold increase and 87±11% inhibition). This finding supports the notion that PS and nicastrin both are present in active γ-secretase.

## Discussion

γ-Secretase is a potential target for AD treatments, since it cleaves the amyloid precursor protein (APP) to generate the neurotoxic Aβ peptide. However, clinical trials with γ-secretase inhibitors have shown disappointing results, mainly due to toxic side effects caused by inhibited γ-secretase processing of other substrates than APP, for instance Notch. To successfully treat AD by inhibiting γ-secretase, selective alteration of the enzymatic activity would be required. It is therefore necessary to considerably enhance the understanding about which factors that regulate the various γ-secretase activities and where in the cell these activities take place. Future approaches to design AD treatments could thus be, for instance, to target γ-secretase at only specific subcellular locations.

We have taken a new approach to study γ-secretase by developing a highly sensitive method to differentiate between active and inactive γ-secretase in situ. To this end, we designed GTB that binds only to active γ-secretase and contains additional groups that allow the inhibitor to be visualized by PLA. The similarity in IC50 values for the inhibition of Aβ formation for GTB (11.3 nM) and L-685,458 (∼10–20 nM) [14], [17] suggests that the inhibitor part of GTB is unaffected by the introduction of additional groups in the molecule. Similarly, the finding that PS1-CTF, PS1-NTF, Aph-1 and nicastrin were immunopurified with GTB demonstrates that it efficiently binds to active γ-secretase. PLA assays require several incubations and washing steps. Therefore, it is important that GTB contains a photoreactive group that becomes covalently bound to neighboring proteins upon UV irradiation and remains attached to those proteins, even after extensive incubation and many washing steps. The method was evaluated by comparing the association between nicastrin and PS, using either PS and nicastrin antibodies to visualize all PS-nicastrin associations in the cells, or GTB and nicastrin antibody to visualize only active γ-secretase.

Proximity ligation assays used to study the association between γ-secretase components or their subcellular locations have not previously been reported. In this study, we have used PLA with three different setups; i) single recognition setup to evaluate antibodies and distribution of proteins; ii) PLA with two different primary antibodies for protein-protein association studies and iii) our novel method using GTB for active enzyme-protein association studies. Evaluation of the PS1 and nicastrin antibodies with PLA served both to study whether the antibodies bind to the γ-secretase components in the hippocampal neurons and to show how PS1 and nicastrin are distributed in the neurons. The high density of PLA signals observed throughout the entire neuron is consistent with the reported subcellular localization of γ-secretase components in several compartments, including the nuclear membrane, ER/Golgi, mitochondria-associated membranes (MAMs), synaptic vesicles, endosomes and the plasma membrane [25], [26], [27]. It was only the staining in the nuclei that was somewhat surprising. However, it has previously been suggested that PS1 is localized to the nuclei during certain stages of mouse brain development [28]. In addition, it should be noted that PLA is a highly sensitive method that allows single molecule detection and, thus, is capable of detecting proteins that traditional immunostaining methods are not sufficiently sensitive to detect.

The traditional PLA setup employs two primary antibodies and two PLA probes, and it has, based on the size of the antibodies and oligoucleotides been reported that molecules with binding distances of 40 nm or less can be detected [29]. GTB is expected to reach by at most ∼5 nm out from the membrane and the diameter of streptavidin tetramers is estimated to about 4–5 nm, and hence, we estimate that molecules associated with a binding distance of ∼30 nm or less will give rise to a signal in our novel assay. The reported EM 3D structures of γ-secretase suggest that the width of the complex is between 7–12 nm [11], [12], [13]. In line with this notion, the interactions between proteins within this complex are detectable, both by using PLA assays with only antibodies and with GTB and antibodies.

The selectivity of the inhibitory component of GTB (L-685,458) has previously been studied by comparing IC 50 values for γ-secretase and other proteases, showing a ∼60-fold difference compared to the aspartyl protease HIV-1, and even higher differences compared to other types of proteases [14]. In the GTB-proximity ligation method developed here, the specificity is increased, due to the fact that proximity ligation requires two detection probes. One detection probe was directed to GTB and the other to primary antibodies to PS or nicastrin. Our novel method thus secures the specificity of GTB for γ-secretase to a level beyond the specificity of the inhibitor component on its own.

The number of PLA signals obtained when the PS antibody was used in pair with the nicastrin antibody was around 900/cell. The corresponding number when PS antibody was replaced by GTB was ∼200, but assuming that only the portion that was competed by L-685,458 is specific and should be counted, this number was ∼150 signals/cell. Thus, these data indicate that 17% of the nicastrin-PS associations occur with mature γ-secretase. Notably, the neurons were fixed and permeabilized under mild conditions (4% formalin for 10 min at RT and 0.4% CHAPSO for 10 min at RT, respectively). At this CHAPSO concentration, γ-secretase activity is preserved [30], and since GTB was capable of binding to γ-secretase, the active site appears to be intact. There is a risk, however, that the proportion of active γ-secretase/PS is somewhat underestimated, for instance due to the formaldehyde fixation.

Another interesting observation was that there were relatively fewer PLA signals in the nuclear/perinuclear region in the PLA assay using the nicastrin antibody and GTB than in the assay using the nicastrin and PS antibodies. Both the finding that the interactions between active γ-secretase and nicastrin constitute only a portion of the total amount of PS1-nicastrin interactions, and that the PLA signals obtained by the GTB assay were located more peripherally compared to the PLA assay using only antibodies, are in line with the notion that γ-secretase needs to mature to become active. It has not, however, been established where the maturation occurs. Our data with the GTB method thus gives novel information as to where γ-secretase matures, which appears to be distal from the perinuclear ER system.

In conclusion, we present a novel proximity ligation-based method to visualize active γ-secretase. By comparing the results from this method with traditional PLA in neurons, we enable differentiation of active from inactive γ-secretase in the cells. The technique is highly sensitive and allows detection of single complexes. Since the assay is conducted in situ, it allows determination of the subcellular location of active enzymes as well as their association with other proteins. The selectivity of the assay for active enzymes is achieved by using an active site inhibitor of γ-secretase, which is conjugated to a PEG linker, a photoreactive group and a biotin group. The assay has dual specificity because it requires both an active site and an additional γ-secretase component to give a signal. With all these advantages over more traditional techniques for studying membrane protein interactions, this method opens the doors for a multitude of applications. For instance, by combining the GTB assay with subcellular marker staining and high resolution microscopy, such as STED, a detailed analysis of the subcellular location of the active enzyme complexes could be done. Moreover, we propose that similar assays could be used to study protein-protein interactions for and subcellular location of other enzymes.

## Supporting Information

Figure S1 **Staining of neurons with GTB in the absence or presence of L-685,458.** Mouse primary hippocampal neurons were stained with 200 nM GTB and fluorescently labeled SA as described in Materials and Methods in the absence (A) or presence (B) of 10 µM L-685,458.
(TIF)

Figure S2 **PLA using nicastrin and PS1 antibodies in cells lacking PS1 and PS2.** PLA was conducted with anti-PS1-NTF and mouse anti-Nct-CT, as described in Materials and Methods, in BD 3 cells (A) and BD8 cells that are deficient in PS1 and PS2 (B).
(TIF)
