# Supplementary figures and images for: Visualizing Active Enzyme Complexes Using a Photoreactive Inhibitor for Proximity Ligation – Application on γ-Secretase
Source: PLoS One. 2013 May 24;8(5):e63962. doi: 10.1371/journal.pone.0063962 (PMC3663845; doi:10.1371/journal.pone.0063962)

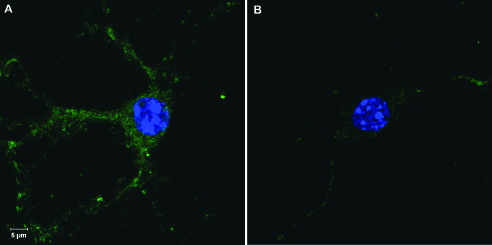

Supplement: Figure S1 — Staining of neurons with GTB in the absence or presence of L-685,458. Mouse primary hippocampal neurons were stained with 200 nM GTB and fluorescently labeled SA as described in Materials and Methods in the absence (A) or presence (B) of 10 µM L-685,458. (TIF) [file pone.0063962.s001.tif]

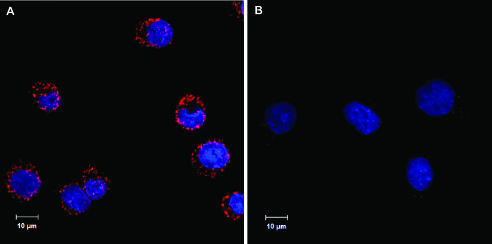

Supplement: Figure S2 — PLA using nicastrin and PS1 antibodies in cells lacking PS1 and PS2. PLA was conducted with anti-PS1-NTF and mouse anti-Nct-CT, as described in Materials and Methods, in BD 3 cells (A) and BD8 cells that are deficient in PS1 and PS2 (B). (TIF) [file pone.0063962.s002.tif]
